# Global transcriptome profiling reveals differential regulatory, metabolic and hormonal networks during somatic embryogenesis in *Coffea arabica*

**DOI:** 10.1186/s12864-022-09098-z

**Published:** 2023-01-24

**Authors:** Rayan Awada, Maud Lepelley, David Breton, Aline Charpagne, Claudine Campa, Victoria Berry, Frédéric Georget, Jean-Christophe Breitler, Sophie Léran, Doâa Djerrab, Federico Martinez-Seidel, Patrick Descombes, Dominique Crouzillat, Benoît Bertrand, Hervé Etienne

**Affiliations:** 1 Nestlé Research - Plant Science Research Unit, Tours, France; 2 UMR DIADE, CIRAD, Montpellier, France; 3 UMR DIADE, Université de Montpellier, CIRAD, Montpellier, IRD France; 4 Nestlé Research, Société Des Produits Nestlé SA, Lausanne, Switzerland; 5 Sophia Genetics, Genève, Switzerland; 6 UMR DIADE, IRD, Montpellier, France; 7 Max Planck Institute for Molecular Plant Physiology, Golm, Germany; 8 School of BioSciences, The University of Melbourne, Parkville, VIC Australia

**Keywords:** Cell fate, Coffee, Molecular markers, Molecular networks, Somatic embryogenesis, Totipotency, Transcriptomics

## Abstract

**Background:**

Somatic embryogenesis (SE) is one of the most promising processes for large-scale dissemination of elite varieties. However, for many plant species, optimizing SE protocols still relies on a trial and error approach. We report the first global scale transcriptome profiling performed at all developmental stages of SE in coffee to unravel the mechanisms that regulate cell fate and totipotency.

**Results:**

RNA-seq of 48 samples (12 developmental stages × 4 biological replicates) generated 90 million high quality reads per sample, approximately 74% of which were uniquely mapped to the Arabica genome. First, the statistical analysis of transcript data clearly grouped SE developmental stages into seven important phases (Leaf, Dedifferentiation, Primary callus, Embryogenic callus, Embryogenic cell clusters, Redifferentiation and Embryo) enabling the identification of six key developmental phase switches, which are strategic for the overall biological efficiency of embryo regeneration. Differential gene expression and functional analysis showed that genes encoding transcription factors, stress-related genes, metabolism-related genes and hormone signaling-related genes were significantly enriched. Second, the standard environmental drivers used to control SE, i.e. light, growth regulators and cell density, were clearly perceived at the molecular level at different developmental stages. Third, expression profiles of auxin-related genes, transcription factor-related genes and secondary metabolism-related genes were analyzed during SE. Gene co-expression networks were also inferred. Auxin-related genes were upregulated during dedifferentiation and redifferentiation while transcription factor-related genes were switched on from the embryogenic callus and onward. Secondary metabolism-related genes were switched off during dedifferentiation and switched back on at the onset of redifferentiation. Secondary metabolites and endogenous IAA content were tightly linked with their respective gene expression. Lastly, comparing Arabica embryogenic and non-embryogenic cell transcriptomes enabled the identification of biological processes involved in the acquisition of embryogenic capacity.

**Conclusions:**

The present analysis showed that transcript fingerprints are discriminating signatures of cell fate and are under the direct influence of environmental drivers. A total of 23 molecular candidates were successfully identified overall the 12 developmental stages and can be tested in many plant species to optimize SE protocols in a rational way.

**Supplementary Information:**

The online version contains supplementary material available at 10.1186/s12864-022-09098-z.

## Introduction

Somatic embryogenesis (SE) is a developmental process in which a plant somatic cell can dedifferentiate into a totipotent embryogenic stem cell that has the ability to redifferentiate into an embryo and give rise to a true-to-type plant under appropriate culture conditions [1–3]. Since its first description in carrot [4, 5], this process has been reported in a wide range of both annual [6–8] and perennial plant species [9–13]. SE has been shown to have major advantages when applied to forest tree species enabling clonal mass propagation, cryopreservation of valuable germplasm and genetic transformation [14, 15]. SE is particularly useful for plants with a long life cycle (woody species) and are difficult to propagate using conventional horticultural methods like cuttings [16].

The ability of a somatic cell to undergo embryogenesis in vitro is both an inherent and an acquired characteristic that requires just the right combination of genotype, explant type, explant source and the culture environment [17]. The most efficient treatments used to induce SE vary, ranging from the application of exogenous growth regulators to abiotic stress. Under the appropriate conditions, the explant produces differentiated embryos, either directly from the explant or indirectly from a callus [18]. Direct SE is often described as a low-yield method and indirect SE as a high-yield method [10]. The morphological and cellular changes that occur during in vitro embryogenesis are well-described in the literature [1, 19, 20]. Briefly, nine developmental stages have been characterized in the indirect SE of dicots: explant, primary then embryogenic callus, embryogenic cell clusters, pro-embryogenic masses, globular embryos, heart-shaped embryos, torpedo-shaped embryos and cotyledonary embryos, before developing into a whole plant. In contrast to the detailed knowledge available on morphological and histological events, little is known about the molecular mechanisms underlying the successful transition between the different developmental stages that occur during SE and in the expression of totipotency.

Research on SE remains mainly empirical, characterized by a low-throughput trial-and-error approach. A set of drawbacks have been reported, especially a strong genotypic effect, difficulty in obtaining embryogenic calli, low quality of regenerated embryos, and more generally, the lack of efficiency of certain steps [12, 21, 22] leading to hitherto prohibitive production costs and overall slow technical progress. Although SE has already been widely described in a number of woody species [9–11, 13], propagating adult woody plants remains an arduous, labor intensive, and tricky operation. Lack of knowledge on the mechanisms underlying the reprogramming of somatic cells is the main obstacle to improving SE processes [23, 24].

Many authors consider that applying cutting-edge omics technologies to SE would tremendously impact our knowledge of the underlying molecular mechanisms [24–26]. Indeed, transcriptomics can provide a wealth of information for the description and elucidation of physiological responses to environmental conditions in plants [27]. However, to date, few authors have applied an RNA-seq approach to SE and most studies have focused on the early events of SE induction in annual plants such as Arabidopsis [28], cotton [29], rice [30], maize [31]. In woody plants, Gautier et al. [32] compared embryogenic callus (EC) and non-embryogenic callus (NEC) in Douglas-fir (*P. menziesii*), and more recently, Chen et al. [33], Wang et al. [34] and Qi et al. [35] compared EC and redifferentating embryos in *Dimocarpus longan*, *Hevea brasiliensis* and hybrid sweetgum (*Liquidambar styraciflua* × *Liquidambar formosana*) respectively.

Coffee is one of the world’s favorite beverages. It has a major economic impact on many producing countries, especially in South America and Vietnam [36]. Today, SE applied to coffee is one of the most advanced technologies in plant mass vegetative propagation [24]. Thirty years of research on coffee SE has led to the successful large-scale dissemination of *Coffea arabica* F1 hybrids and *C. canephora* cv. Robusta clones [22]. The biological efficiency of the processes established in the two cultivated species is largely successful allowing to produce high-yielding and time-synchronized independent cell lines [37–40]. However, the production costs associated with SE remain high and still cannot compete with the production costs of seedlings or traditional cuttings [41].

Indeed, like in other species, coffee SE research remains empirical and characterized by slow technical progress. For example, 10 years of laborious research were needed to develop culture conditions for the mass redifferentiation of embryogenic clusters into somatic embryos in liquid nutrient media [24]. Some SE developmental switches are considered real black boxes due to the lack of knowledge about the cellular and molecular events involved. Current production cannot meet increasing market demand estimated at 50–100 million coffee vitro plants per year, and a scale-up is urgently needed [24]. Based on a detailed knowledge of associated molecular mechanisms, rational optimization now seems possible.

In this paper, we draw transcript profiles of 12 SE developmental stages, from leaf explant dedifferentiation until formation of globular embryos. In addition to the developmental stages previously described by Verdeil et al. [1], we decided to sample the dedifferentiation episode in order to have a more continuous sampling. A robust statistical method based on transcript modulations was used to identify the main developmental switches and biological processes involved. Differentially expressed genes (DEGs) involved in three of the biological processes identified i.e. genes encoding transcription factors, genes related to phytohormone biosynthesis and response, and genes encoding secondary metabolites, were then studied more deeply. Co-expression networks between these gene families were also revealed. Lastly, comparing Arabica embryogenic and non-embryogenic calli enabled the identification of biological processes involved in the acquisition of the embryogenic capacity.

## Results

### High sampling quality enabled high read reliability between replicates

The availability of efficient large-scale propagation protocols for coffee SE, currently used at the commercial level, enabled us to place more than 1,000 leaf explants on dedifferentiation medium in each of the four replicates (Fig. S1A) as well as to establish a total of 20 independent embryogenic cell lines (Fig. S1B). The quality of sampling was validated by the fact that all the resulting cell lines were high yielding and time synchronized during embryo regeneration (Fig. S1C). A three-dimensional PCA allowed to check the reliability between the four biological replicates at each developmental stage based on the normalized expression values of all 41,569 genes (Fig. S1D).

### Clustering DEG profiles divided the Arabica SE process into seven developmental phases

**Fig. 1:**
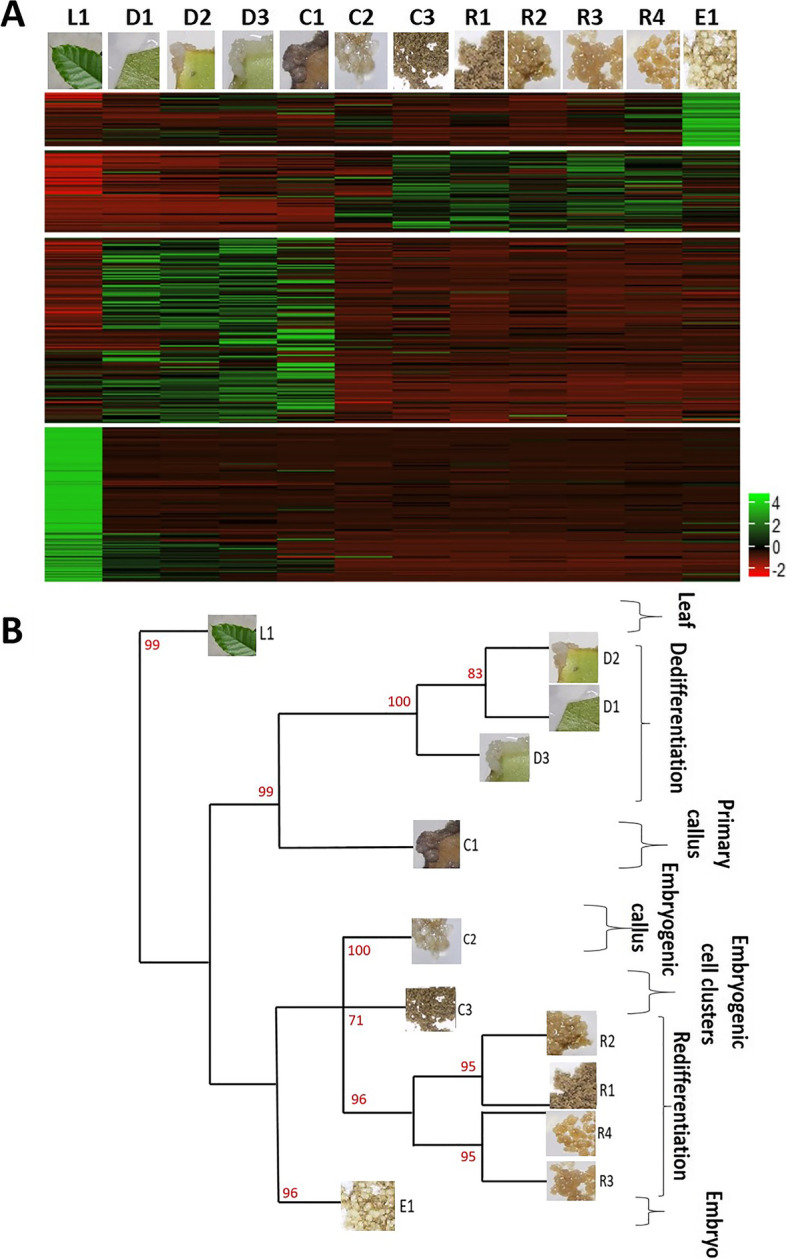
Profiling of differentially expressed genes during 12 key developmental stages of Arabica somatic embryogenesis (SE). **A** Heatmap generated from the normalized counts of 10,384 DEGs over the 12 developmental stages. Rows correspond to DEGs and columns to the developmental stages. Normalized counts of each gene were transformed in order to follow a standard normal distribution. Positive expression values are in green and negative values in red. **B** Hierarchical clustering of the 12 SE developmental stages according to the similarities in their transcript profiles. Clustering was performed using Pearson’s correlation coefficient. Cluster probabilities were calculated via a multiscale bootstrap with a total of 1,000 iterations. Clustering yielded 7 major nodes: Leaf, Dedifferentiation, Primary callus, Embryogenic callus, Embryogenic cell clusters, Redifferentiation and Embryo

High resolution analysis of differentially expressed genes (DEGs) during SE was performed using RNA-seq technology. A total of 10,384 DEGs were obtained over the 12 SE developmental stages (Table S1). The heatmap generated from the normalized counts of the total DEGs over the 12 SE developmental stages showed similar transcript profiles in some of these stages (Fig. 1A). Bootstrapped hierarchical clustering analysis was performed to highlight stages that shared similar transcript profiles (Fig. 1B). Strong correlations between profiles were obtained resulting in seven major nodes: the “Leaf” node including the L1 stage transcript profile; the “Dedifferentiation” node in which the D1, D2 and D3 stages clustered together; the “Primary Callus”, “Embryogenic callus” and “Embryogenic cell clusters” nodes in which the C1, C2 and C3 stages clustered distinctly; the “Redifferentiation” node in which the R1, R2, R3 and R4 stages clustered together; and the “Embryo” node in which the E1 stage transcript profile differed from the redifferentiation stages. The obtained clusters revealed a developmental pattern as the nodes corresponded to successive developmental phases of the SE process, i.e., the leaf phase, the leaf explant dedifferentiation phase, the primary callus phase, the embryogenic callus phase, the embryogenic cell clusters phase, the redifferentiation phase (from embryogenic cell clusters to embryoid structures), and the embryo phase (globular embryos).

### Transcriptional characterisation of six major developmental phase switches during Arabica SE

**Fig. 2:**
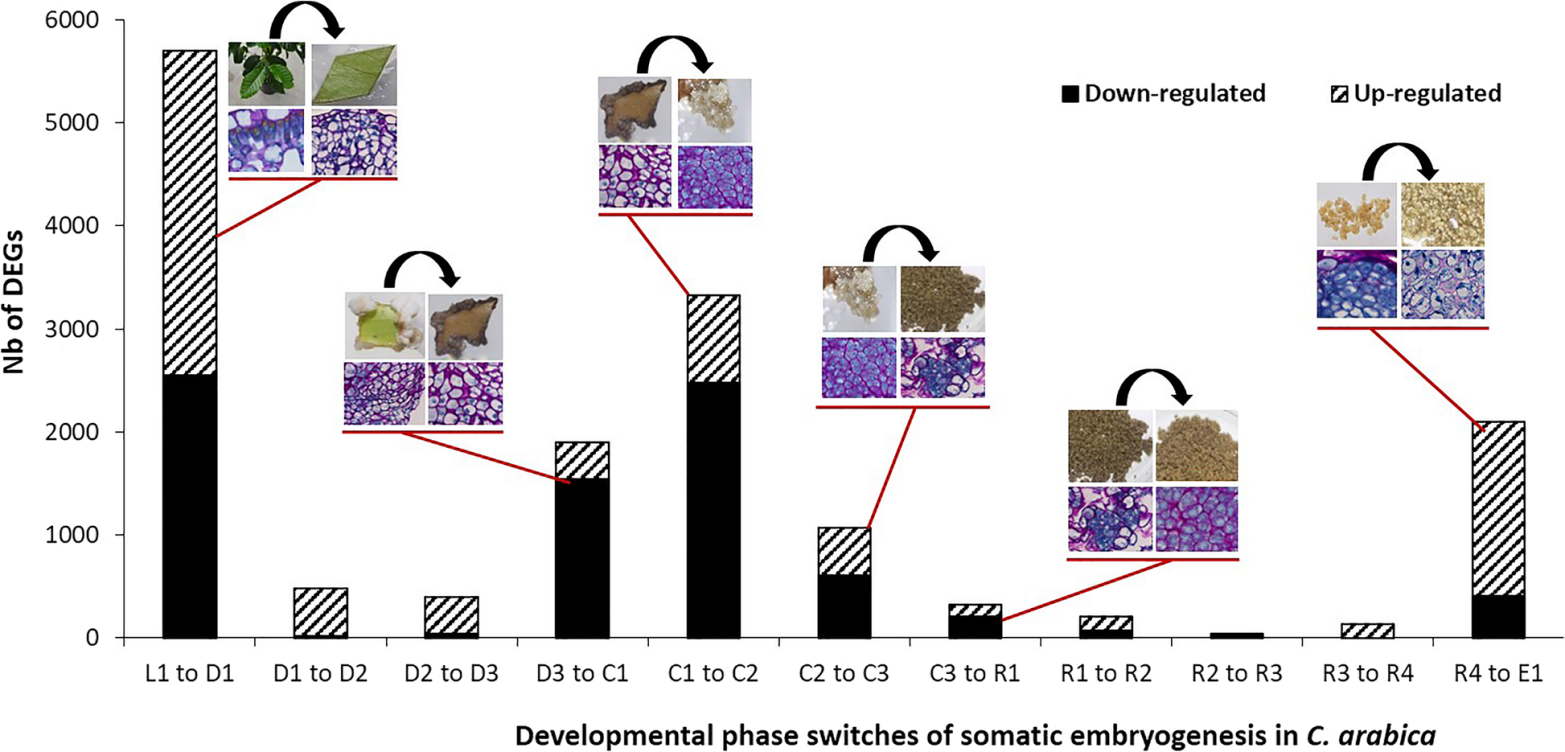
Distribution of differentially expressed genes among key developmental phase switches during Arabica somatic embryogenesis (SE). Upregulated (striped) and downregulated (black) gene numbers are given for 12 developmental stages of the Arabica SE process. Red lines correspond to the identified developmental phase switches: Leaf-to-Dedifferentiation (L1 to D1), Dedifferentiation-to-Primary callus (D3 to C1), Primary callus-to-Embryogenic callus (C1 to C2), Embryogenic callus-to-Embryogenic cell clusters (C2 to C3), Embryogenic cell clusters-to-Redifferentiation (C3 to R1) and Redifferentiation to Embryo (R4 to E1)

The number of genes up- or downregulated at different developmental stages is shown in Fig. 2. Based on the hierarchical clustering analysis, the 12 studied developmental stages were grouped into seven developmental phases (Fig. 1B). The number of DEGs was particularly high during the transition from one developmental phase to another, thus characterizing a developmental phase switch. In chronological order, these six switches were (Fig. 2): Leaf-to-Dedifferentiation (L1 to D1), Dedifferentiation-to-Primary callus (D3 to C1), Primary callus-to-Embryogenic callus (C1 to C2), Embryogenic callus-to-Embryogenic cell clusters (C2 to C3), Embryogenic cell clusters-to-Redifferentiation (C3 to R1) and Redifferentiation to Embryo (R4 to E1). The highest number of DEGS was found (5,701) during the first phase switch (L1 to D1), 55% of these genes were upregulated and 45% were downregulated. The four next switches, i.e. D3 to C1, C1 to C2, C2 to C3 and C3 to R1, had 1,894; 3,326; 1,074 and 323 DEGs respectively, and at least 75% of the genes were downregulated in each of these switches. The last developmental phase switch (R4 to E1) had 2,098 DEGs of which 80% were upregulated. It is interesting to note that the passages from D1 to D3 that characterize early stages of dedifferentiation do not appear to be remarkable switches. This is also the case for R1 to R4 stages that characterize redifferentiation and where no remarkable switches occurred either.

**Fig. 3:**
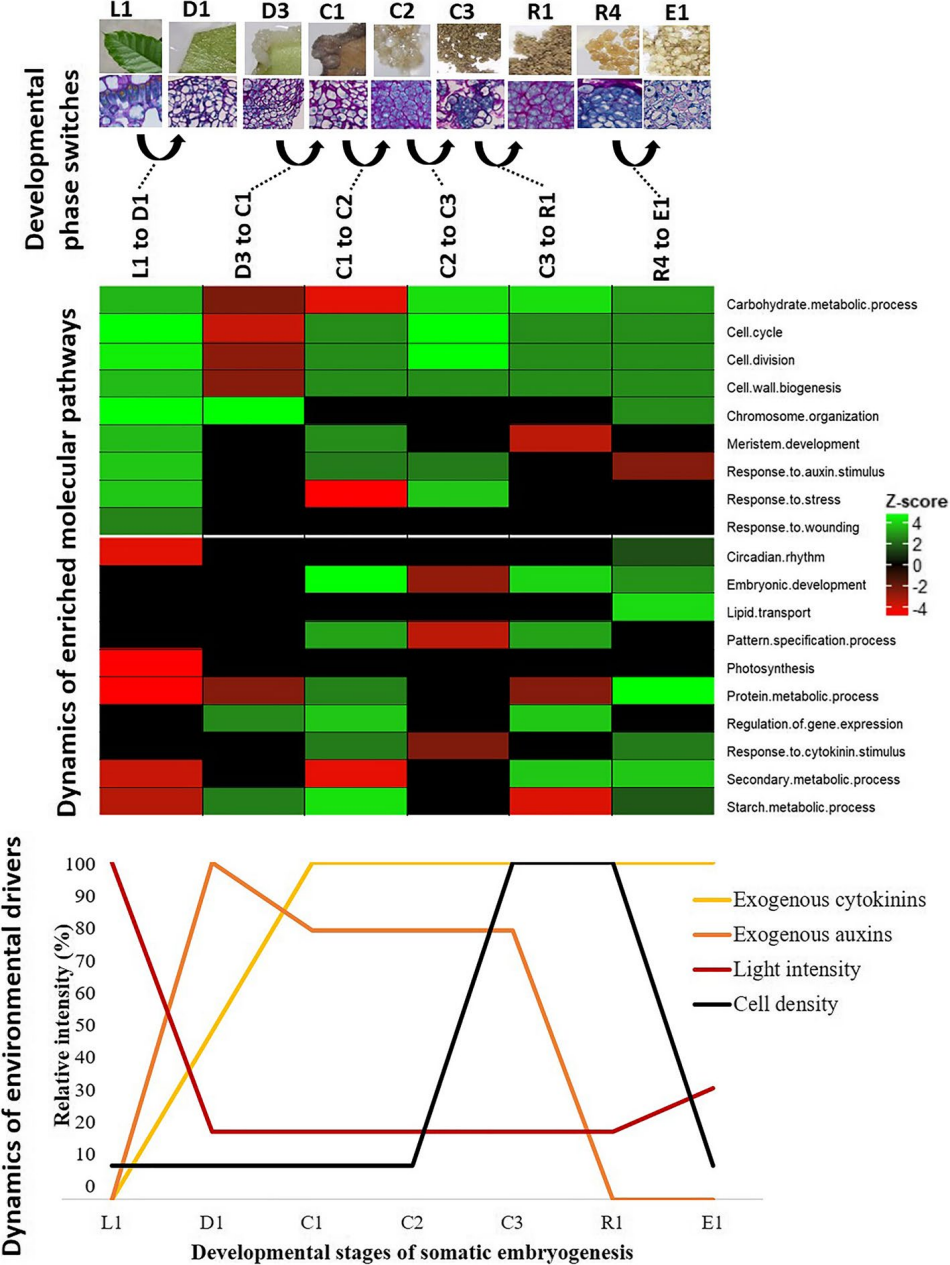
Functional categorization of the Arabica differentially expressed genes (DEGs) during the six key developmental switches. DEGs were first compared against The Arabidopsis Information Resource database (TAIR, www.arabidopsis.org) using BLASTP with an e-value cut-off of 1 × 10^−4^. Functional categorization of Arabidopsis orthologs in all 6 developmental phase switches was performed using the Parametric Analysis of Gene set Enrichment (PAGE) tool in agriGO v2.0. The PAGE tool yielded significant gene ontology (GO) terms that were classified in 19 biological processes. Rows correspond to biological processes and columns to the developmental phase switches. Positive Z-score values are in green and negative values in red. A schematic representation of the dynamics of environmental drivers during the Arabica SE process is provided under the heatmap. The intensity levels of the environmental drivers are expressed as their relative intensity

DEGs for the six key developmental phase switches were compared against The Arabidopsis Information Resource database (TAIR, www.arabidopsis.org) using BLASTP with an e-value cut-off of 1 × 10^−4^. Approximately 50% of the *C. arabica* DEGs had an Arabidopsis ortholog since the *C. arabica* species is an allotetraploid originated from two different wild diploid ancestor species (2n = 22), C. *canephora* and C. *eugenioides* [42]. The PAGE tool yielded significant gene ontology (GO) terms that were classified into 19 biological processes (Fig. 3). Each process was sharply upregulated or downregulated at the different developmental switches. These processes can be regrouped into six families: hormonal pathways (mainly auxin and cytokinin), metabolic pathways (carbohydrate, starch, protein and secondary metabolism), regulatory pathways (regulation of gene expression, pattern specification, embryo development), stress-related pathways (response to stress and wounding), mitosis-related pathways (cell cycle and division, cell wall and chromatin organization), and photosynthesis-related pathways (photosynthesis and circadian cycle).

### Effects of environmental drivers on the regulation of biological processes during SE

SE involves a number of exogenous artificial factors that are perceived as environmental cues by plant cell or tissue cultures. These environmental drivers (light, temperature, exogenous growth regulators mainly auxins and cytokins, cell density) were widely reported as factors influencing the success of the SE process [8, 12]. In this study, variations in these drivers made it possible to study their influence on gene expression and, in so doing, to check whether they are important drivers. In Fig. 3, we show that environmental drivers are tightly linked to the regulation of genes involved in crucial biological processes. For example, at the beginning of the SE process, light exposure was suppressed through a transition from a 12 h/12 h photoperiod in the greenhouse (L1) to obscurity in the culture chamber (D1) before being restored to partial light conditions during globular embryo formation (E1). Variations in light intensity were directly linked to variations in the expression of genes involved in photosynthesis and circadian rhythm. Similarly, genes involved in response to the auxin and cytokinin stimuli followed the application or removal of these exogenous hormones in their respective medium. In parallel, high cell density also plays a crucial role, mainly during the proliferation of embryogenic cell clusters, as it inhibits the embryonic pathway and enhances biomass proliferation. This was clearly reflected in the downregulation of genes involved in embryonic development and pattern specification processes in embryogenic cell clusters, while upregulation of the same genes occurred when cell density decreased sharply during redifferentiation. Genes involved in the response to wounding were also upregulated during the transition from entire leaves (L1) to dissected leaf squares (D1) needed to build the explant and induce the dedifferentiation mechanisms.

### Analysis of genes encoding transcription factors during Arabica SE

**Fig. 4:**
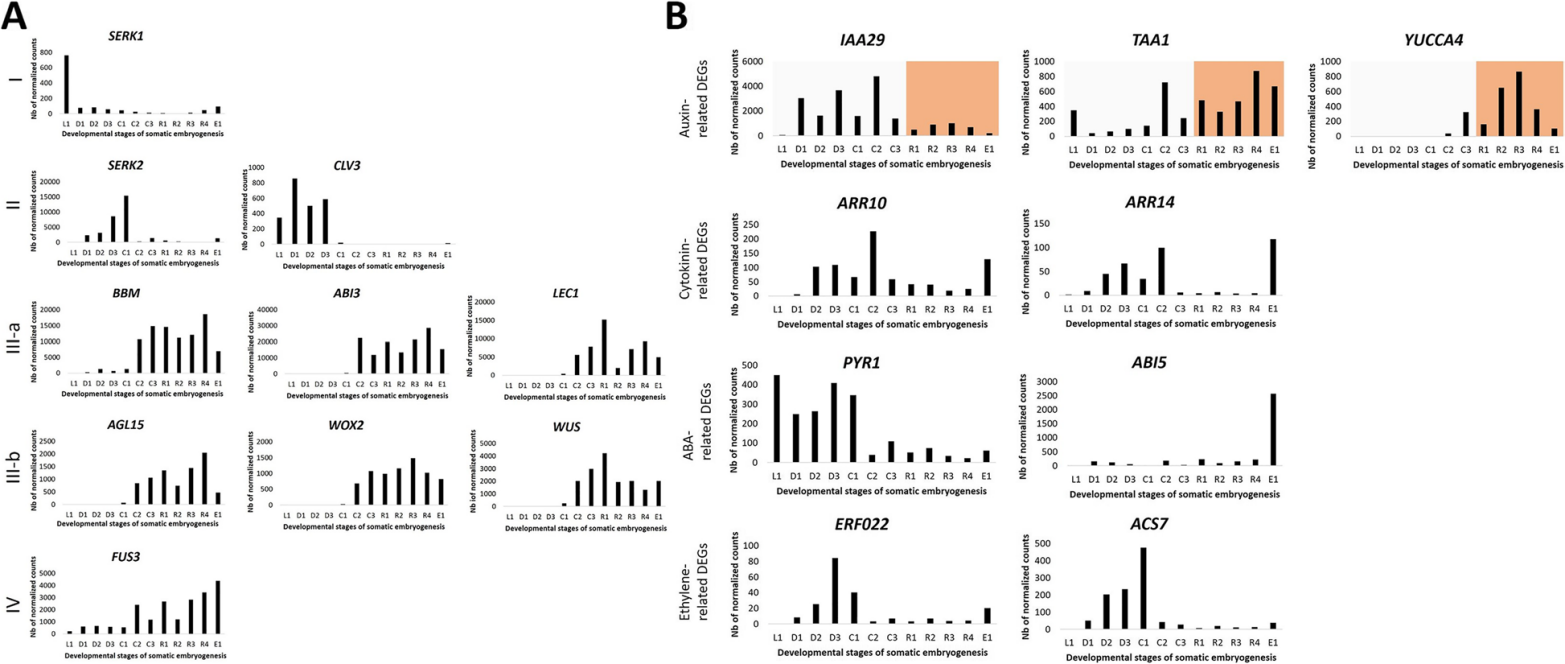
Expression profiles of transcription factor-encoding genes and hormone-related genes during key stages of Arabica SE. The x-axis corresponds to the different developmental stages and the y-axis to the number of normalized counts for each gene after RNA-seq read mapping on the Arabica reference genome followed by DESEq2 normalization. **A** *SERK1*, *SERK2*, *CLV3*, *BBM*, *ABI3*, *LEC1*, *AGL15*, *WOX2*, *WUS* and *FUS3* were studied. Genes were classified in 4 types of expression profiles (I, II, III, IV). Class III was subdivided into 2 subclasses (a & b) corresponding to high and low gene expression, respectively. **B** Auxin-related genes (*IAA29*, *TAA1* and *YUCCA4*), cytokinin-related genes (*ARR10*, *ARR14*), ABA-related genes (*PYR1*, *ABI5*) and ethylene-related genes (*ERF022*, *ACS7*) were investigated. The colored area corresponds to total exogenous auxin removal from the medium

Many authors reported the involvement of certain transcription factors (TFs) in the induction of somatic embryos in different species, including abscisic acid (ABA) INSENSITIVE 3 (*ABI3*) [43, 44], AGAMOUS LIKE 15 (*AGL15*) [45, 46], BABY BOOM (*BBM*) [47, 48], LEAFY COTYLEDON (*LEC1/LEC2*) [49, 50], WUSCHEL-RELATED HOMEOBOX (*WUS/WOX2*) [51, 52], SOMATIC EMBRYOGENESIS RECEPTOR KINASE (*SERK*) [53, 54], CLAVATA 3 (*CLV3*) [55] and FUSCA 3 (*FUS3*) [56]. Figure 4A shows the detailed kinetics of these genes throughout the *C. arabica* SE process. Four types of expression profiles were obtained: (i) *SERK1* was highly active in the differentiated cells belonging to leaf tissues and decreased sharply during the initial leaf-to-dedifferentiation phase switch (L1 to D1); (ii) *CLV3* was upregulated at the first dedifferentiation stage (D1) while *SERK2* was switched on at the same stage and gradually upregulated to reach the maximum in the primary callus cells. *SERK2* had a high expression level while *CLV3* had a low expression profile. Both genes were switched off during the primary callus-to-embryogenic callus switch, and hence were not expressed in embryogenic cells, embryogenic cell clusters or embryos; (iii) *BBM*, *ABI3*, *LEC1*, *AGL15*, *WOX2* and *WUS* expression was induced during the primary callus-to-embryogenic callus switch and remained expressed in embryogenic cells, cell clusters and pro-embryos. These six genes were further classified into two groups: highly expressed genes (*BBM*, *ABI3*, *LEC1*) and lowly expressed genes (*AGL15*, *WOX2*, *WUS*); (iv) *FUS3* was expressed during the whole SE, first at a low level during the first five developmental stages, then at a high level in embryogenic tissues and during the embryo redifferentiation phase, with the highest expression in globular embryos (E1).

### Analysis of genes encoding hormone signaling pathways during somatic embryogenesis

The role of hormones in the induction and maturation of somatic embryos has been widely reported, in particular, that of auxin, cytokinin, abscisic acid and ethylene [57]. As we previously studied the dynamics of these endogenous hormones during the different key steps of SE [58], in the present work we took the opportunity to analyze the kinetics of some hormone-related DEGs during the same SE key developmental steps (Fig. 4B). *Aux/IAA* repressor genes (*IAA29*, *IAA30*, *IAA31*) have been reported to contribute to SE induction [59]. Here, we showed that the most highly expressed gene in *C. arabica* was *IAA29*. This gene was induced at the beginning of the dedifferentiation phase after exogenous auxin was added in the medium, and reached its highest level of expression in embryogenic calli. Logically, this gene was downregulated when the exogenous auxin was removed from the medium but was still expressed at a low level during the redifferentiation phase leading to the formation of the embryo.

**Fig. 5:**
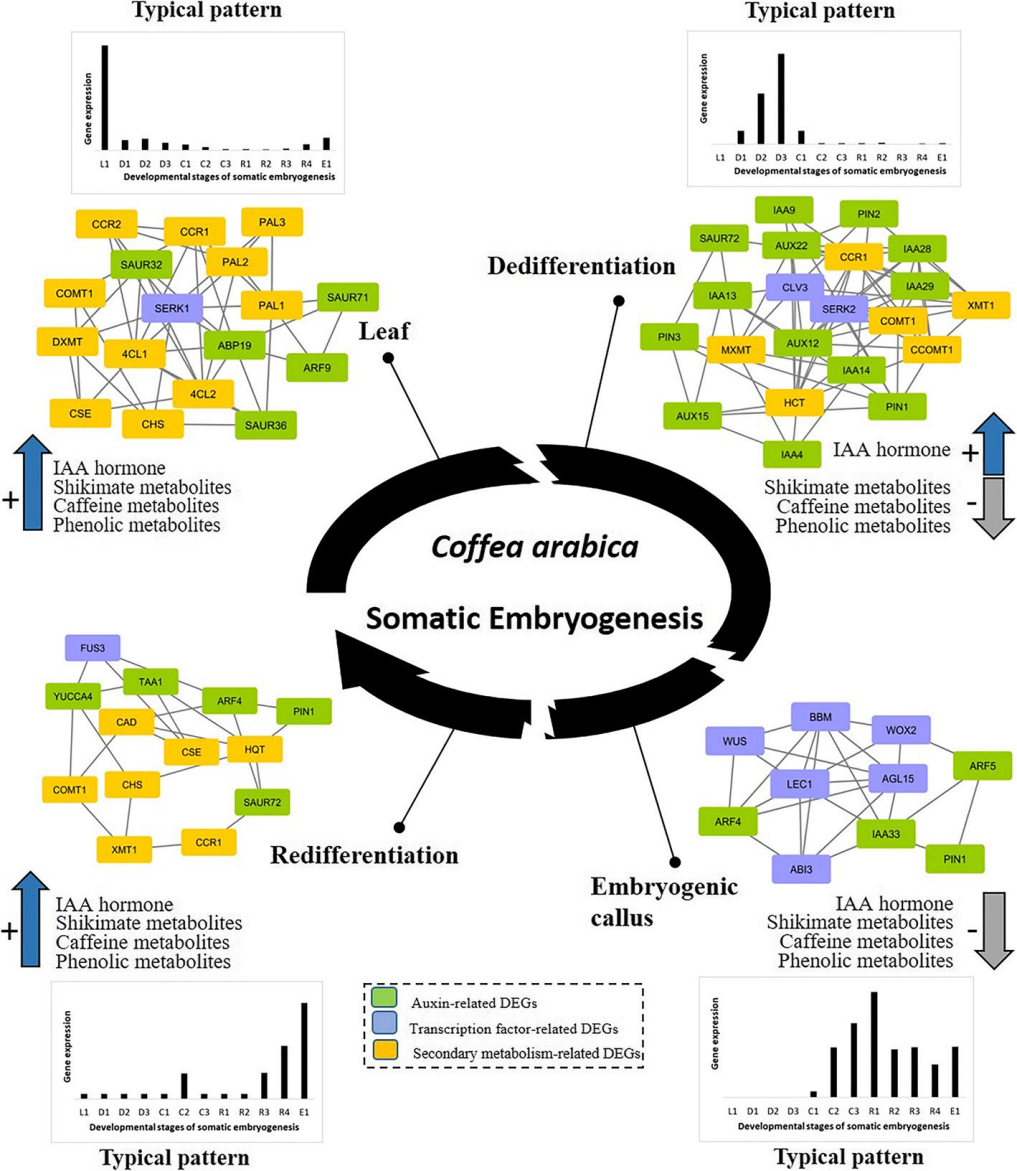
Co-expression analysis of auxin-, TFs- and secondary metabolism-related DEGs during the four main SE phases. Four clusters of genes were generated corresponding to the four developmental phases: Leaf, Dedifferentiation, Embryogenic callus and Redifferentiation. Auxin-related genes are in green, transcription factor-related genes in violet and secondary metabolite-related genes in orange. An average expression profile of the genes present in each cluster was also generated (bar diagrams). For each cluster, a gene co-expression network was inferred using the ARACNE algorithm. All the networks were visualized in Cytoscape. Co-expressed genes share edges. Schematic kinetics of IAA and secondary metabolite endogenous content are also presented (Fig. S2)

Both *Tryptophan Aminotransferase* of *Arabidopsis* (*TAA*) and *YUCCA* family genes are needed in the tryptophan-dependent indole-3-acetic acid (IAA) biosynthesis pathway [60]. TAA plays a role in the conversion of tryptophan to indole-3-pyruvic acid (IPA) and YUCCA is involved in the conversion of IPA to IAA. In *C. arabica* cells, *TAA1* and *YUCCA4* were the most highly expressed genes in their respective gene families. Our results show that *TAA1* is highly upregulated in embryogenic callus cells and during redifferentiation particularly after removal of exogenous auxin (from the R1 stage on) followed by *YUCCA4*, which is highly upregulated in R2 and R3 stages, indicating auxin biosynthesis in developing embryos. *YUCCA4* is also active in embryogenic cell clusters probably due to an insufficient amount of exogenous auxin in the medium. Transcript profiling revealed that auxin-related genes differed in their expression profiles, as further detailed in Fig. 5.

Cytokinin-response regulators like *Arabidopsis Response Regulators* (*ARRs*) [61], were also investigated. Two type-B *ARRs, ARR10* and *ARR14,* were differentially expressed during *C. arabica* SE. Both genes had a similar expression profile. They were induced during dedifferentiation and their expression reached maximum in embryogenic calli (C2) before they were strongly downregulated during the redifferentiation phase until embryo formation when they were both again sharply upregulated. *WUSCHEL*, whose expression was strongly upregulated in embryogenic callus cells (Fig. 4A), has been reported to be a direct target of *ARR10* in *Arabidopsis* [62].

The *PYR1* gene encodes a protein that plays a major role in the ABA receptor PYR/PYL/RCAR [63]. Our results showed that *PYR1* was highly expressed in the leaf during the dedifferentiation phase and in the primary callus, and was further sharply downregulated in the embryogenic callus and during the further redifferentiation stages. Downstream genes encoding TFs of the ABA response pathway were induced in embryogenic callus (*ABI3*, Fig. 4A) or later during embryo formation (*ABI5*, Fig. 4B).

*Ethylene Responsive Factor* 022 (*ERF022*) has been reported to promote the formation of somatic embryos in Arabidopsis through the ethylene-related pathway and to negatively regulate *1-aminocyclopropane-1-Carboxylate Synthase 7* (*ACS7*) involved in ethylene biosynthesis in Arabidopsis [64]. Our results showed that *ERF022* was highly activated during the dedifferentiation phase and was also upregulated during the embryo formation stage (E1) and downregulated the expression of *ACS7* when expressed in *C. arabica*. Ethylene has been reported to inhibit the formation of somatic embryos when present in the culture medium [64]. *ACS7* reached its highest expression in the primary callus and was strongly downregulated in embryogenic callus, while *BBM*, a gene that also encodes an ethylene-responsive TF, was induced in embryogenic callus (Fig. 4A).

### Auxin, transcription factors and secondary metabolism pathways are highly modulated during somatic embryogenesis

Among the different biological processes that take place in SE (identified in Fig. 3), we chose to focus on three: auxin, transcription factors and secondary metabolism pathways, since their variation appears to play a fundamental role in the SE process. All auxin-related DEGs, SE transcription factor-related DEGs, and secondary metabolism-related DEGs were selected for cluster analysis. For didactic purposes, we generated four clusters, each cluster corresponding to a type of expression profile. The four clusters obtained were divided into four typical patterns (Fig. 5): (i) Genes that were highly active during the leaf phase and sharply downregulated during dedifferentiation, (ii) genes that were highly upregulated during dedifferentiation and sharply downregulated during the embryogenic callus phase, (iii) genes induced during the embryogenic callus phase, (iv) genes that were upregulated during redifferentiation and embryo formation. A gene co-expression network was inferred for each cluster. The four resulting networks (Leaf, Dedifferentiation, Embryogenic callus and Redifferentiation) are summarized in Table 1. In addition to transcriptomic data, schematic kinetics of IAA and secondary metabolite endogenous contents are presented in Fig. S2.

**Table 1:** Generated gene co-expression networks and genes belonging to each network

Co-expression network	Genes belonging to the co-expression network		
	Transcription factor-related genes	Auxin-related genes	Secondary metabolism-related genes
Leaf	*SERK1*	*Auxin Response Factor 9 (ARF9)* *Small Auxin Up RNA 32 (SAUR32)* *SAUR36* *SAUR71* *Auxin Binding Protein 19 (ABP19)*	*Phenylalanine Ammonia Lyase 1 (PAL1)* *PAL2* *PAL3* *4-cinnamoyl CoA ligase 1 (4CL1)* *4CL2* *Chalcone Synthase (CHS)* *Caffeine Synthase (DXMT)* *Caffeoyl Shikimate Esterase (CSE)* *Cinnamoyl* ***-*** *CoA Reductase 1 (CCR1) CCR2* *Caffeic acid O-MethylTransferase (COMT1)*
Dedifferentiation	*SERK2* *CLV3*	*AUX12* *AUX15* *AUX22* *IAA4* *IAA9* *IAA13* *IAA14* *IAA28* *IAA29* *PIN-FORMED1 (PIN1)* *PIN2* *PIN3* *SAUR72*	*Xanthosine Methyltransferase 1 (XMT1)* *MXMT* *CCR1* *COMT1* *Caffeoyl-CoA-O-MethylTransferase 1 (CCOMT1)* *HydroxyCinnamoyl-CoA shikimate/quinate hydroxycinnamoyl Transferase (HCT)*
Embryogenic callus	*ABI3* *BBM* *WUS* *WOX2* *AGL15* *LEC1*	*ARF4* *ARF5* *IAA33* *TAA1*	
Redifferentiation	*FUS3*	*ARF4* *TAA1* *YUCCA4* *SAUR72* *PIN1*	*CHS* *XMT1* *CSE* *CCR1* *COMT1* *Hydroxycinnamoyl-CoA Quinate hydroxycinnamoyl Transferase (HQT) Cinnamyl-Alcohol Dehydrogenase (CAD)*

Auxin-related genes, transcription factor-related genes and secondary metabolism-related genes clustered in four types of expression profiles and co-expression networks were inferred. Four co-expression networks were obtained: Leaf, Dedifferentiation, Embryogenic callus, Redifferentiation

Eleven genes related to secondary metabolism were expressed in leaf cells and defined the “Leaf” network. On a metabolic level, shikimate, caffeine and phenolic metabolites accumulated in leaves indicating a tight link between gene expression and metabolite content. Five genes involved in endogenous auxin response were also expressed in leaf cells, and endogenous IAA was also present. Among the genes encoding SE-related TFs, only *SERK1* was expressed in leaf cells. *SERK1* appeared at the center of the gene co-expression network as it shared the most edges with other genes (Fig. 5).

The “Dedifferentiation” network was mainly composed of auxin-related genes (13 genes) that were upregulated during the dedifferentiation phase while six genes related to secondary metabolism were also expressed during this phase. Similarly, endogenous IAA was over-accumulated compared to in the leaf phase while shikimate, caffeine and phenolic metabolites were under-accumulated (Fig. 5). Precursors of these metabolites over-accumulated, indicating probable inhibition in their respective biosynthesis pathways. *SERK2* and *CLV3,* two genes encoding SE-related TFs, were upregulated during the dedifferentiation phase and appeared at the center of the gene co-expression network as they shared the most edges with other genes.

The “Embryogenic callus” network was a reduced network, mostly composed of genes encoding TFs and four auxin-related genes. The edges were equally shared between genes encoding TFs. No secondary metabolism-related DEGs were upregulated in this network. During this phase, endogenous IAA as well as shikimate, caffeine and phenolic metabolites under-accumulated (Fig. 5) confirming the tight relation between genes and metabolites.

The “Redifferentiation” network was shared equally between secondary metabolism-related genes and auxin-related genes (Fig. 5). *TAA1* and *YUCCA4* were also upregulated indicating activation of endogenous auxin biosynthesis. This was confirmed by the levels of endogenous IAA that were over-accumulated compared to the levels of IAA in embryogenic callus. Similarly, secondary metabolites were over-accumulated again compared to their levels in embryogenic callus and tended to reach the same levels as in leaf cells. *FUS3* was upregulated during the redifferentiation phase and was directly linked to *TAA1* and *YUCCA4,* indicating a role in the activation of endogenous auxin biosynthesis leading to embryo formation.

### Embryogenic and non-embryogenic calli differ strongly at the transcriptomic level

**Fig. 6:**
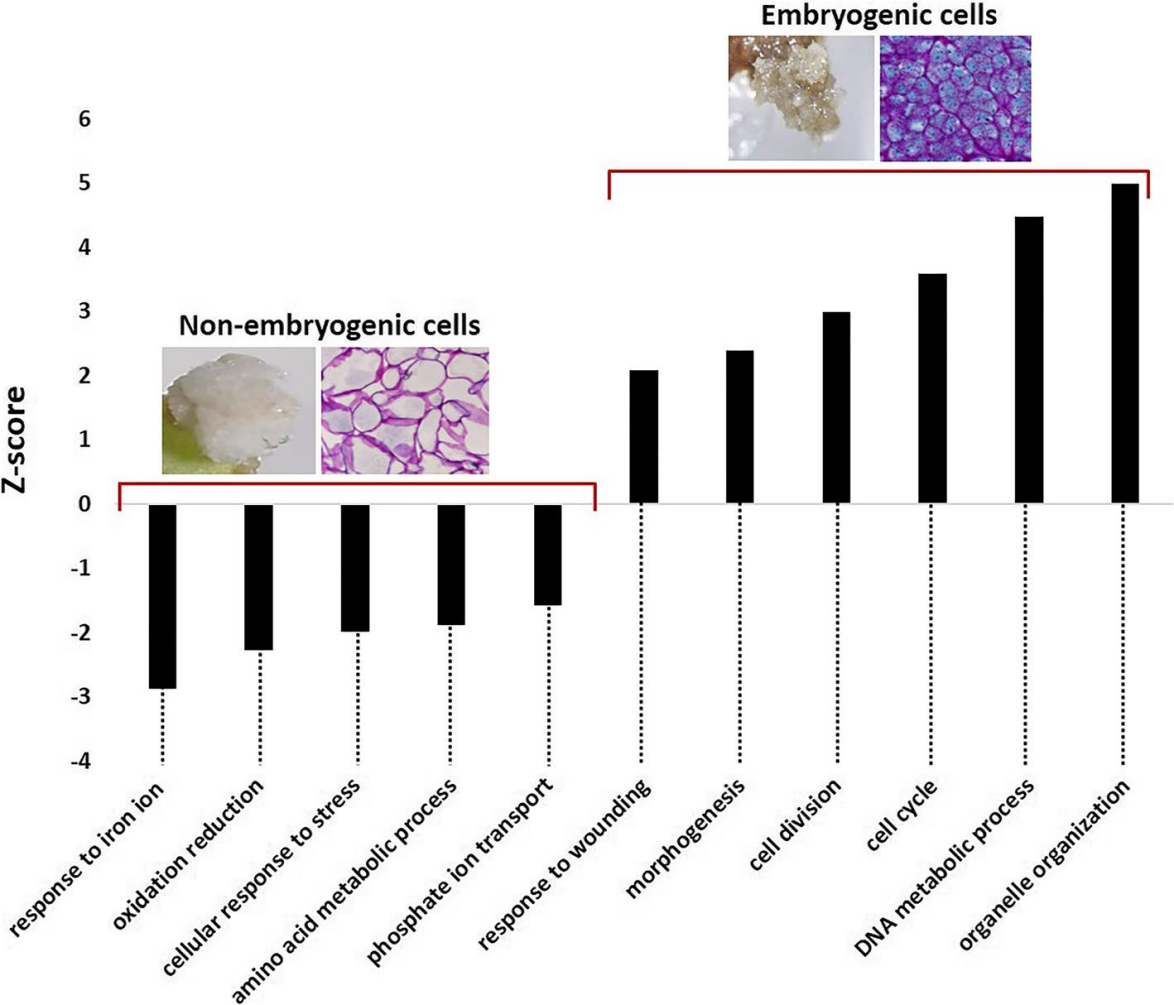
Functional categorization of Arabica differentially expressed genes (DEGs) in embryogenic calli compared to non-embryogenic calli. An additional stage, the non-embryogenic callus (NEC), was sampled at the same time as the embryogenic callus (C2). DEGs were compared against The Arabidopsis Information Resource database (TAIR, www.arabidopsis.org) using BLASTP with an e-value cut-off of 1 × 10^−4^. Functional categorization of Arabidopsis orthologs was performed using the Parametric Analysis of Gene set Enrichment (PAGE) tool in agriGO v2.0 . The PAGE tool yielded significant gene ontology (GO) terms that were classified in 11 biological processes. The x-axis corresponds to the different biological processes obtained and the y-axis corresponds to the Z-score values yielded by the PAGE tool

Why some of the thousands of cells evolve into an embryogenic callus while others proliferate undifferentiated is a fundamental question. Our study allowed us to address this issue. We previously showed that embryogenic (EC) and non-embryogenic calli (NEC) can easily be distinguished on the basis of their morphology, color and cell characteristics [58]. It was not possible to regenerate somatic embryos from NEC. At the transcriptomic level, 346 DEGs were obtained between embryogenic cells and non-embryogenic cells (Table S2). Surprisingly, most of these genes (305/346 DEGs) were downregulated in embryogenic cells compared to non-embryogenic cells while only 41 were upregulated. The PAGE tool yielded significant GO terms that were classified as 11 different biological processes (Fig. 6). Compared to non-embryogenic cells, in embryogenic cells the PAGE tool showed downregulation of genes encoding processes mainly including response to iron ion, oxidation reduction, cellular response to stress, amino acid metabolic process and phosphate ion transport while it showed upregulation of genes involved in organelle organization, DNA metabolic process, cell cycle and division, morphogenesis and response to wounding.

## Discussion

### Expression profiles of somatic embryogenesis–specific genes as a signature of cell fate

This paper reports on one of the first global analyses of SE gene expression on 12 key developmental stages covering the regeneration process from leaf explant dedifferentiation to embryo formation. Two pre-requisites were crucial for this study: (i) the availability of large-scale SE protocols, offering biological efficiency and cell homogeneity at each developmental stage, (ii) the availability of recent omics technologies. This global analysis of coffee SE could serve as a reference for a wide range of plant species because the intensive sampling of successive key developmental stages that are conserved among species provided an overview of the SE process and enabled us to open some real black boxes [24]. The statistical approach we used to analyze transcriptomic data allowed us to cluster the whole SE process in seven main developmental phases and six key developmental phase switches that are the basis of SE. Many authors recently reported the need for a better understanding of the SE process to remove existing bottlenecks [8, 22, 24, 26]. Many developmental stages are undistinguishable when conventional morphological and histological approaches (e.g. early stages of dedifferentiation and redifferentiation) are used. A number of studies in different species assumed that clear correlations exist among the different transcriptome profiles and certain SE stages [28, 29, 31]. Our global analysis is a proof of concept that transcripts are good markers of all cell fate transitions and, in the near future, could be used to understand and better pilot the optimization of the SE culture conditions by using them as a milestone of successful developmental stages. This goes beyond morphological and histological descriptions, which until now, were the most common way to support empirical protocol optimization. We believe that this global scale transcriptome study, combined with a metabolic approach, will lead to a much clearer understanding of the molecular mechanisms underlying cell reprogramming.

### Genes encoding regulatory, metabolic, hormonal and stress-related pathways are the most differentially expressed during coffee somatic embryogenesis

A number of genes were strongly up or downregulated during the six developmental phase switches identified. The transition of a leaf cell to an embryogenic cell is a long process of cell division and organization that occurs in the dark, hence upregulated genes related to mitosis and downregulated genes related to photosynthesis were expected [65]. SE is driven by exogenously supplied plant growth regulators [66]. Although most plants require similar physical conditions (temperature, light regime) for the induction of SE, only a specific composition of the medium can trigger and subsequently support the process. According to Sugimoto et al. [67], the prime characteristic of plant regeneration is cell fate reprogramming induced by wounding, stress, and hormones, in agreement with our results. Auxins and cytokinins are widely known to play essential roles in the induction of embryogenic culture [22, 57, 58, 68, 69]. Additionally, many authors have reported the involvement of certain transcription factors (TFs) in the induction of somatic embryos in different species. However, the time points at which the genes encoding these TFs are highly active were previously unknown. In this study, we showed the kinetics of these genes during the successive SE steps for the first time. It has been proposed that together with auxin and cytokinin, TFs play a crucial role in the maintenance of the stem cell niche in the shoot apical meristem in totipotent cells in Arabidopsis [70] and in the cell pattern specification during the transition from totipotent-to-embryonic cell at the onset of redifferentiation [71]. Our results confirmed that an exogenous supply of auxin and cytokinin enabled enrichment in the transcripts of genes related to meristem development in coffee embryogenic cells, while enrichment in transcripts of genes related to embryonic cell pattern specification and embryonic formation was evidenced at the onset of redifferentiation. Concerning metabolic pathways, we previously showed that carbohydrates, starch, amino acids and secondary metabolites are differentially accumulated during the different SE steps [58]. Here, we confirmed that DEGs involved in metabolic pathways are tightly linked to the accumulation of their respective metabolites.

### Genes regulating cell fate are highly modulated by environmental drivers during somatic embryogenesis

This study provides solid proof that environmental drivers are the main regulators of cell fate as they are tightly linked to the regulation of genes involved in crucial biological processes. This study allowed us to measure the direct effects of the environmental drivers usually used to control SE, particularly light, growth regulators, and cell density [8, 12] as these drivers were clearly perceived by the cells at molecular level. Environmental drivers, conventionally named ‘culture conditions’, are usually optimized in an empirical way to guarantee the appropriate nutritional and physico-chemical environment for a particular genotype for SE induction [22, 24]. Since these drivers can be perceived at a molecular level, the genes modulated by the drivers are of huge interest since they can be used to pilot SE optimization in a rational way. This is the case for genes involved in photosynthesis and circadian rhythm that are tightly linked to light intensity and photoperiod, genes involved in the response to auxin and cytokinin stimulus, and genes involved in embryo pattern specification that are tightly linked to cell density. Many authors have focused on the complex gene networks involved in the response to growth regulators, mainly auxin and cytokinin, to understand the expression of cell totipotency in Arabidopsis and in cotton [8, 71, 72]. The genes identified in these model plants were also found in coffee [68] and showed similar patterns during the developmental stages we studied, indicating conserved pathways of cell totipotency between species. We believe that coffee SE could be used as a reference system to better understand fundamental mechanisms behind the response of woody plants to growth regulators.

### Hormone-related genes play a major role in the expression of totipotency

SE is driven by exogenously supplied plant growth regulators. At the transcriptomic level, the upregulation of a total of 13 *AUX/IAA* genes, which are repressor genes [71] as well as efflux carrier genes (*PIN*), showed that the deprogramming process leading to the formation of undifferentiated cells in *C. arabica* was highly dependent on exogenously supplied auxin. Endogenous IAA concentrations are known to be tightly linked to expression of the *YUCCA* gene in Arabidopsis [73]. In coffee, once auxin was removed from the medium to allow cell redifferentiation, all *AUX/IAA* genes were switched off and *YUCCA* genes were switched on, enabling synthesis of endogenous IAA. Our results confirmed the concomitant increase in *YUCCA* gene expression and in endogenous IAA during redifferentiation.

*ARF5* appeared to be only upregulated in embryogenic cells and can now be considered as a potential molecular marker of this developmental stage in coffee. Wójcikowska et al. [74] and Quintana-Escobar et al. [75] showed that, in Arabidopsis and in *C. canephora* respectively, *ARF5* is highly expressed in embryogenic cultures and regulates the expression of numerous genes involved in somatic embryo formation including *LEC2* (*LEAFY COTYLEDON2*), which is an activator of the *YUC1*, *YUC4*, *YUC10* (*YUCCA*) genes involved in auxin biosynthesis during SE. *ARF5* has also been reported to be involved in the cytokinin response pathway in Arabidopsis, tightly linked to *ARR10* during the patterning and cell organization of meristem cells [74]. We also confirmed upregulation of *ARR10* in coffee embryogenic cells that occurred at the same time as upregulation of the *ARF5* gene. This shows that SE pathways are highly conserved between species and that the upregulation of *ARR10* can be considered as a molecular candidate for embryogenic potential (Table 2).

**Table 2:** Transcriptomic markers characterizing the different developmental phases of coffee somatic embryogenesis

Transcriptomic markers	Developmental phases						
	Leaf cells	Cells in dedifferentiation	Primary callus cells	Embryogenic cells	Embryogenic cell clusters	Cells in redifferentiation	Embryonic cells
Transcription factor-related genes	*SERK1*	*SERK2* *CLV3*		*BBM* *ABI3* *LEC1* *AGL15* *WOX2* *WUS*		*FUS3*	*FUS3*
Auxin-related genes		*AUX/IAA*		*ARF5*	*YUCCA4*	*YUCCA4*	
Cytokinin related genes				*ARR10*			*ARR10*
ABA-related genes							*ABI5*
Ethylene-related genes			*ACS7*				
Secondary metabolism-related genes	*PAL* *CHS* *4CL* *DXMT*					*CHS* *4CL* *CAD* *HQT*	*CHS* *4CL* *CAD* *HQT* *XMT*

*ABI3* has been reported to play a major role in the regulation of SE induction in many species [22, 35, 44, 57], while *ABI5* has been reported to inhibit seed germination and promote embryo maturation in conifers [76, 77]. This shows once again that SE pathways are highly conserved between species and that activation of *ABI3* can be considered as a molecular candidate of embryogenic cells, while the activation of *ABI5* is a potential marker of the embryo maturation process (Table 2).

### Identifying molecular candidates of embryogenic capacity

The formation and proliferation of embryogenic cells are the most crucial stages for the success of the SE process in all plant species because the efficiency of redifferentiation (i.e. mass regeneration of embryos) depends directly on their abundance. Embryogenic cell formation is a real bottleneck in the SE process for all plant species including coffee. It requires improvement of culture conditions to reduce the long time required (7 months for coffee). Consequently, many authors have focused their research on comparing embryogenic and non-embryogenic callus on a morphological [1] or molecular level [8, 32]. Since this question is of interest to many researchers, we decided to add the transcriptomic comparison between embryogenic and non-embryogenic callus to our study. A huge majority of the obtained DEGs (305) were downregulated in embryogenic cells compared to non-embryogenic ones. This is in agreement with Yang et al. [8], who showed in cotton that the existing developmental information of somatic cells must be switched off, most probably by an epigenetic regulation, in order to express the embryogenic capacity. The identified DEGs could serve as predictors of regenerative capacity, i.e. used to rapidly select or eliminate cell lines based on their presence/absence. Since coffee embryogenic callus is a compact, rapidly proliferating structure, its constitutive embryogenic cells show functional mitotic activity, upregulation of genes encoding organelle and DNA organization, morphogenesis, cell cycle and division. In addition, upregulation of genes related to wounding (*WIND* genes) [78] in embryogenic cells suggests that they result from a controlled stress-related pathway, while non-embryogenic cells result from an uncontrolled stress-related pathway implicating strong upregulation of genes involved in stress and oxidation processes [79]. Histological studies demonstrated that non-embryogenic callus is a spongy and oxidated callus containing numerous vacuolated and degenerating cells [58]. Non-embryogenic cells were characterized by upregulation of genes coded to respond to metal ion, oxidation reduction, and phosphate ion transport, in agreement with a number of studies on conifers [13, 32] suggesting that the main fate of non-embryogenic cells was survival, while embryogenic cells were mainly a transient state before the cell fate transition. This implies that the markers of embryogenic state in woody plants are conserved between species. We suggest that reducing oxidative stress by improving gaseous O_2_/CO_2_ exchange and reducing ethylene would increase embryogenic capacity.

In our study, transcripts of genes encoding amino acids were also more abundant in non-embryogenic cells, suggesting that the embryogenic cell genes were involved in the synthesis of more complex structures (proteins, DNA) as reported in other species [29, 32].

### Secondary metabolism-related genes are switched off during dedifferentiation and switched back on at the onset of redifferentiation

Somatic cells in the plant contain all the genetic information needed to create a new complete functional plant [8]. During cell dedifferentiation, the existing developmental information of somatic cells must be switched off or reconfigured to make the somatic cells ready for an embryogenic program [80]. Our results clearly showed that genes encoding phenolic compounds and alkaloids were sharply downregulated during this stage and completely switched off in embryogenic cells. This is in agreement with the huge re-configurations observed in cell metabolic pathways during dedifferentiation [58]. Nic-Can et al. [81] provided evidence that these compounds inhibited the embryogenic process by affecting DNA methylation in *C. canephora*. Magnani et al. [82] also reported that biochemical pathways in Arabidopsis were shut off in order to activate the transcriptional machinery. Conversely, our results showed upregulation of the secondary metabolism-related genes in the early days of redifferentiation followed by the resumption of phenolic compound synthesis, mainly chlorogenic acids, as they are key intermediaries for cell wall biogenesis [58]. Furthermore, these antioxidant compounds could intervene as protectants since embryo formation has been widely reported to be a stress-related phenomenon [80, 83]. Therefore, genes involved in chlorogenic acid synthesis, such as *HQT* or lignin synthesis, such as *CAD* [84], can be considered as potential molecular markers of the redifferentiation pattern of coffee SE. Transcripts of genes encoding precursors of caffeine (*XMT1*) [85] also accumulated during redifferentiation, probably meaning that caffeine is produced in later embryo developmental stages.

### Molecular candidates of cell fate to pilot optimization of somatic embryogenesis

We identified a set of potential molecular markers of cell fate transition during coffee SE (summarized in Table 2). A molecular marker is first defined by a clear expression pattern, i.e. a gene is switched on (or sharply upregulated) then switched off (or sharply downregulated) at one or several developmental stages. Secondly, a molecular marker is chosen based on its gene expression level, i.e. a gene with a high expression level is preferred to a gene with a low expression level. Finally, a molecular marker can be validated by comparing it between non-optimal conditions or developmental stages. To be able to undertake detailed sampling of all developmental stages of the coffee SE process, we limited this study to only one genotype. Further RT-qPCR analyses of a set of genotypes that are more or less recalcitrant to the induction of somatic embryos, will be crucial to study the candidate molecular markers. Molecular markers can be used to efficiently pilot the SE process optimization more rapidly, reliably, and more cost-effectively by testing a number of contrasted culture conditions in order to select the optimal ones, particularly in the case of recalcitrant genotypes or species.

## Conclusion

One of the first global scale transcriptome analysis of SE in coffee showed that transcriptomics using the RNAseq technology is a powerful approach to investigate global transcript patterns. This approach clearly identified seven important developmental phases with very contrasted and specific patterns, leading to the characterization of six key developmental phase switches that are strategic for the biological efficiency of embryo regeneration. Using this global transcriptome profiling approach, we proved that transcriptomics can assign a specific signature to each developmental stage and hence provide valuable information about cell fate. It also allowed us to measure at the molecular level and for each developmental phase, the direct effects of environmental drivers, particularly the light and growth regulators used to control the regeneration process. Together with the metabolomics approach [58], this study led to a clearer understanding of the intimate mechanisms governing totipotency and SE. It provides a starting point for optimizing coffee SE protocols in a rational way. The 23 transcriptomic candidates we identified, which are specific to the different developmental phases, including the strategic ‘embryogenic state’, should be validated in recalcitrant genotypes. Once validated, they can be used as targets to pilot SE optimization.

## Methods

### Tissue culture and sampling

An intraspecific hybrid (GPFA116) of Ethiopian origin, produced in the Nestlé Arabica breeding program, was used in this study. SE was performed in the Nestlé Research laboratories (Tours, France) based on the large-scale protocols described previously for *C. arabica* [10], with 4 replicates i.e., four independent explant collections in April, June, October and December 2016 from multiple 1-year-old mother plants grown in the Nestlé Research greenhouse. Explants were first cultured in Petri dishes on T1 ‘dedifferentiation 1’ medium, i.e. Murashige and Skoog (MS) half-strength solid medium supplemented with 0.5 mg/L 2,4-D (2,4-dichlorophenoxyacetic acid), 1 mg/L IBA (indole 3-butyric acid) and 2 mg/L 2iP (N^6^-(2-Isopentenyl) adenine) for 1 month before transfer on T2 ‘dedifferentiation 2’ medium, i.e. MS/2 solid medium supplemented with 1 mg/L 2,4-D and 4 mg/L BA (6-benzylaminopurine) for 6 months until the formation of embryogenic calli. Petri dishes were placed at 25 °C in the dark. Embryogenic calli were then inoculated at a rate of 10 g/L in 250-ml Erlenmeyer flasks containing M ‘proliferation’ liquid nutritive medium, i.e. medium supplemented with 0.3 mg/L 2,4-D and 1 mg/L BA and cultured for 4 months on shakers (120 rpm) at 25 °C in the dark, until proliferation of cell clusters. To stimulate regeneration of early somatic embryos, cell clusters were transferred to 250-mL Erlenmeyer flasks containing DIF ‘redifferentiation’ liquid medium lacking the auxin 2,4-D at a density of 10 g/L medium for 1 week then at 1 g/L for 4 weeks until formation of globular embryos. Erlenmeyer flasks were placed on shakers (120 rpm) at 25 °C under indirect light (120–150 µE.m^−2^.s^−1^) during redifferentiation.

Twelve sampling stages were chosen to cover the SE process from leaf dedifferentiation until torpedo-shaped embryos developed as shown in Fig. S3: leaves from greenhouse plants (L1), explants during dedifferentiation [1 week (D1), 2 weeks (D2), 5 weeks (D3)], compact primary callus obtained 3 months after induction (C1), embryogenic callus obtained 7 months after induction (C2), established cell clusters obtained after 4 months in liquid proliferation medium (C3), pro-embryogenic masses [1 week in redifferentiation medium after auxin withdrawal (R1), 24 h in redifferentiation medium after reducing cell density (R2), 72 h (R3), 10 days (R4)] and globular embryos obtained after 3 weeks of culture (E1). An additional stage, non-embryogenic callus (NEC), was also sampled at the same time and in the same culture conditions as the embryogenic callus (C2). Approximately 1 g of fresh weight/sample/replicate was collected for transcriptome analysis and instantly placed in liquid nitrogen before being stored at -80 °C until further analysis. A detailed morphological and cellular characterization of the different developmental stages is given in Awada et al. [58].

### RNA extraction

Frozen tissues were ground to a fine powder in liquid nitrogen and total RNA was extracted using the RNeasy plant Mini Kit (QIAGEN, Germantown, MD—USA) and treated with RNase free DNaseI (QIAGEN) according to the manufacturer’s instructions. The quality and quantity of total RNA were analyzed using the Agilent 2100 Bioanalyzer RNA chip (Agilent Technologies Inc., Santa Clara, CA—USA). The RNA samples with an RNA integrity number (RIN) higher than 7.0 were selected and used for subsequent analyses.

### Illumina sequencing

RNA sequencing was carried out by Nestlé Research (Lausanne, Switzerland). The cDNA libraries were generated using the TruSeq Stranded mRNA Kit (Illumina), followed by PCR amplification for sequencing on Illumina HiSeq 2500. Paired-end cDNA libraries were generated from all samples, and sequencing was performed to generate the ~ 125 bp paired-end reads. FastQC software (v0.11.5) was used for quality control, and assessment of raw Illumina reads in FASTQ format to obtain per base quality, guanine-cytosine (GC) content, and sequence length distribution. Low-quality reads, adapters, and poly-N-containing reads were removed from the raw data. Approximately 95% of high quality reads were obtained in each library from generated data. An average of 90 million paired-end reads was obtained for each library.

### Read mapping and differential gene expression analysis

The pre-processed reads were aligned to the *C. arabica* genome sequence [86] using the STAR (v2.5.3a) software. The uniquely mapped reads to each gene locus were quantified with a maximum of 10 mismatches per paired-end alignment using the Partek E/M algorithm originally described by Xing et al. [87]  and principal component analysis (PCA) was performed to check the homogeneity of the replicates. On average, 74% of sequenced reads per sample were uniquely mapped to the reference genome [86]. DESeq2 [88] was used to standardize reads across libraries and for differential expression analysis. Differential expression was considered at a threshold value of FDR ≤ 0.001 and the absolute value of log2Ratio ≥ 3. A heatmap of all differentially expressed genes was generated in R (https://www.R-project.org) using the ComplexHeatmap package [89]. The same data were used for hierarchical clustering analysis performed with the pvclust package [90] using Pearson’s correlation coefficient. Cluster probabilities were calculated via a multiscale bootstrap with 1,000 iterations. A cluster probability is a percentage that indicates how strongly the cluster is supported by data.

### Functional gene expression analysis

All differentially expressed genes (DEGs) were compared against The Arabidopsis Information Resource database (TAIR, www.arabidopsis.org) using BLASTP with an e-value cut-off of 1 × 10^−4^. The resulting annotation was used to analyze gene ontology (GO) using the Parametric Analysis of Gene set Enrichment (PAGE) tool in agriGO v2.0 with default functions [91]. Significant GO terms were found using the default FDR < 0.05 cutoff value. Obtained Z-scores were plotted on a heatmap generated in R (https://www.R-project.org) using the ComplexHeatmap package [89] or in a bar diagram in Excel.

### Gene co-expression analysis and network construction

A cluster analysis was performed on auxin-related DEGs, SE transcription factor-related DEGs and secondary metabolism-related DEGs. Hierarchical clustering was accomplished by combining pvclust [90] mediated bootstrapping using the k-means method with Pearson’s correlation distance between DEG expression profiles. Four clusters representing the four main types of expression profiles were generated. For genes in each cluster, the ARACNE algorithm [92] was used to infer the gene co-expression network. The ARACNE procedure starts by assigning to each pair of nodes (pair of genes) a weight equal to their mutual information, then all the edges (links between each pair of nodes) are drawn, followed by the removal of the weakest edges based on the assigned weight. All networks were visualized in Cytoscape [93].

### Validation of RNA-seq by RT-qPCR analysis

To validate the RNA-seq study, RT-qPCR experiments were carried out on five SE developmental stages L1, C1, C2, C3, and E1 as previously described by Marraccini et al. [94]. Based on published data, we targeted three of the circadian clock key genes in *C. arabica*: *GI* (Cara019g022520), *LHY* (Cara00s376g005010) and *ELF4* (Cara003g011430), two genes involved in photosynthesis *PORA* (Cara009g016160) and *CAB1* (Cara011g016820), and two genes involved in starch degradation *ISA3* (Cara016g026000) and *GWD1* (Cara021g020800). Primers were designed using Primer3Plus online software (http://www.bioinformatics.nl/cgi-bin/primer3plus/primer3plus.cgi). All reactions were performed in triplicate. The specificity of the PCR products generated for each set of primers was confirmed by analyzing the Tm (dissociation) of amplified products. PCR efficiency (E) was estimated using absolute fluorescence data captured during the exponential phase of amplification of each reaction with the Eq. (1 + E) = 10^(−1/slope)^ [95]. Expression levels were calculated by applying the formula (1 + E)^−ΔΔCt^ where ΔCt _target_ = Ct _target gene_ – Ct _reference gene_ and ΔΔCt = ΔCt _target_ – ΔCt _reference sample_, with the L1 samples being used as references for each construction. Expression levels were normalized by taking the geometric mean of two internal control genes, *24S* (Cara005g012900) and *PP2A* (Cara00s700g005000) [48, 96]. The statistical differences were analyzed by ANOVA based on Fisher’s LSD (*P* < 0.05). No statistical differences were noted (Fig. S4).

## Supplementary Information

**Additional file 1: Figure S1. **The availability of large-scale protocols for coffee somatic embryogenesis (SE) guaranteed reliability and development synchronization at each developmental stage as well as biological efficiency.

**Additional file 2: Figure S2.** Metabolic pathways and hormone dynamics during the four main developmental phase switches.

**Additional file 3: Figure S3. **Characterization of the 12 sampled key developmental stages throughout the Arabica somatic embryogenesis (SE) process at morphological level.

**Additional file 4: Figure S4. **RT-qPCR verification of selected genes in five SE developmental stages L1, C1, C2, C3, and E1.

**Additional file 5: Table S1.** List of differentially expressed genes (DEGs) between the 12 developmental stages covering the SE process (10,384 DEGs) and their normalized counts by DESEq2.

**Additional file 6: Table S2.** List of differentially expressed genes (DEGs) between Embryogenic and Non-embryogenic callus (346 DEGs) and their normalized counts by DESeq2.

## Data Availability

All data generated or analyzed during this study are included in this published article and its supplementary information files. All raw transcriptomic sequence data generated and used to carry out the present study on *Coffea arabica* GPFA116 intraspecific hybrid variety are available at the National Center for Biotechnology Information Sequence Read Archive under accession number PRJNA744419. *Coffea arabica* reference genome (Et39 cultivar) sequencing, assembly and annotation files are available at the National Center for Biotechnology Information Sequence Read Archive under accession number PRJNA698600 (manuscript under publication).

## Abbreviations

2,4-D: 2,4-Dichlorophenoxyacetic acid
2-iP: N^6^-(2-Isopentenyl) adenine
*4CL*: 4-CINNAMOYL COA LIGASE
6-BA: 6-Benzylaminopurine
ABA: *cis*-Abscisic acid
*ABI*: ABSCISIC ACID INSENSITIVE
ACC: 1-Aminocyclopropane-1-carboxylic acid
*ACS*: 1-AMINOACYCLOPROPANE 1-CARBOXYLATE SYNTHASE
*AGL*: AGAMOUS-LIKE
*ARF*: AUXIN RESPONSE FACTOR
*ARR*: ARABIDOPSIS RESPONSE REGULATOR
*BBM*: BABY BOOM
*CAD*: CINNAMYL-ALCOHOL DEHYDROGENASE
*CHS*: CHALCONE SYNTHASE
*CLV*: CLAVATA
*COMT*: CAFFEIC ACID O-METHYLTRANSFERASE
CQA: Caffeoylquinic acid
DEG: Differentially expressed genes
*DXMT*: CAFFEINE SYNTHASE
*EC*: Embryogenic callus
*ERF*: ETHYLENE RESPONSE FACTOR
*FUS*: FUSCA
GO: Gene Ontology
*HQT*: HYDROXYCINNAMOYL-COA QUINATE HYDROXYCINNAMOYL TRANSFERASE
IAA: Indole-3-acetic acid
IBA: Indole 3-butyric acid
*LEC*: LEAFY COTYLEDON
MS: Murashige and Skoog
NEC: Non-embryogenic callus
*PAL*: PHENYLALANINE AMMONIA LYASE
PAGE: Parametric Analysis of Gene set Enrichment
PCA: Principal Component Analysis
*PIN*: PIN-FORMED
*PYR*: PYRABACTIN RESISTANCE
RIN: RNA integrity number
SE: Somatic embryogenesis
*SERK*: SOMATIC EMBRYOGENESIS RECEPTOR KINASE
*TAA*: TRYPTOPHAN AMINOTRANSFERASE
TF: Transcription factor
*WOX*: WUSCHEL-RELATED HOMEOBOX
*WUS*: WUSCHEL
*XMT*: XANTHOSINE METHYLTRANSFERASE
