# Supplementary material for: Global transcriptome profiling reveals differential regulatory, metabolic and hormonal networks during somatic embryogenesis in Coffea arabica
Source: BMC Genomics. 2023 Jan 24;24:41. doi: 10.1186/s12864-022-09098-z (PMC9875526; doi:10.1186/s12864-022-09098-z)
Supplement: Supplementary file 1 — Additional file 1: Figure S1. The availability of large-scale protocols for coffee somatic embryogenesis (SE) guaranteed reliability and development synchronization at each developmental stage as well as biological efficiency. [file 12864_2022_9098_MOESM1_ESM.docx]

**
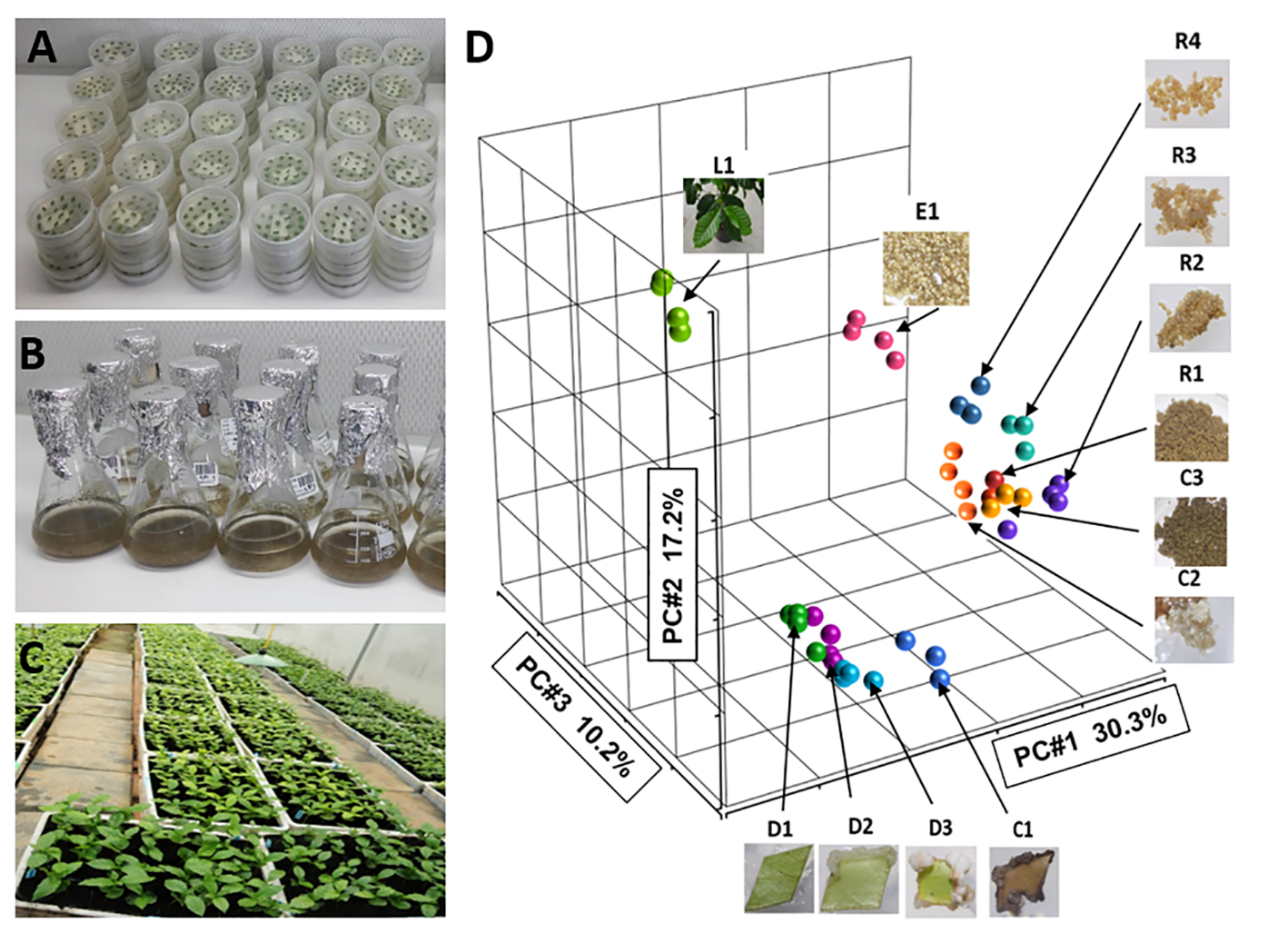
**

**Figure S1.** The availability of large-scale protocols for coffee somatic embryogenesis (SE) guaranteed reliability and development synchronization at each developmental stage as well as biological efficiency. (**A**) More than 1,000 leaf explants per replicate were introduced on dedifferentiation medium for this study. (**B**) Fifty grams of cell clusters were needed to sample the embryogenic cell cluster stage (C3) and the whole redifferentiation episode. (**C**) Embryos were successfully converted into plantlets, thereby validating the quality of all previously sampled stages. (**D**) RNA-seq was carried on 12 developmental stages and a three-dimensional PCA was drawn based on normalized expression values of all 41,569 genes. This analysis confirmed the robustness of the biological replicates, i.e. the four replicates of each stage globally clustered together, and discriminated the different Arabica SE stages.
