# Supplementary material for: Global transcriptome profiling reveals differential regulatory, metabolic and hormonal networks during somatic embryogenesis in Coffea arabica
Source: BMC Genomics. 2023 Jan 24;24:41. doi: 10.1186/s12864-022-09098-z (PMC9875526; doi:10.1186/s12864-022-09098-z)
Supplement: Supplementary file 2 — Additional file 2: Figure S2. Metabolic pathways and hormone dynamics during the four main developmental phase switches. [file 12864_2022_9098_MOESM2_ESM.docx]

**
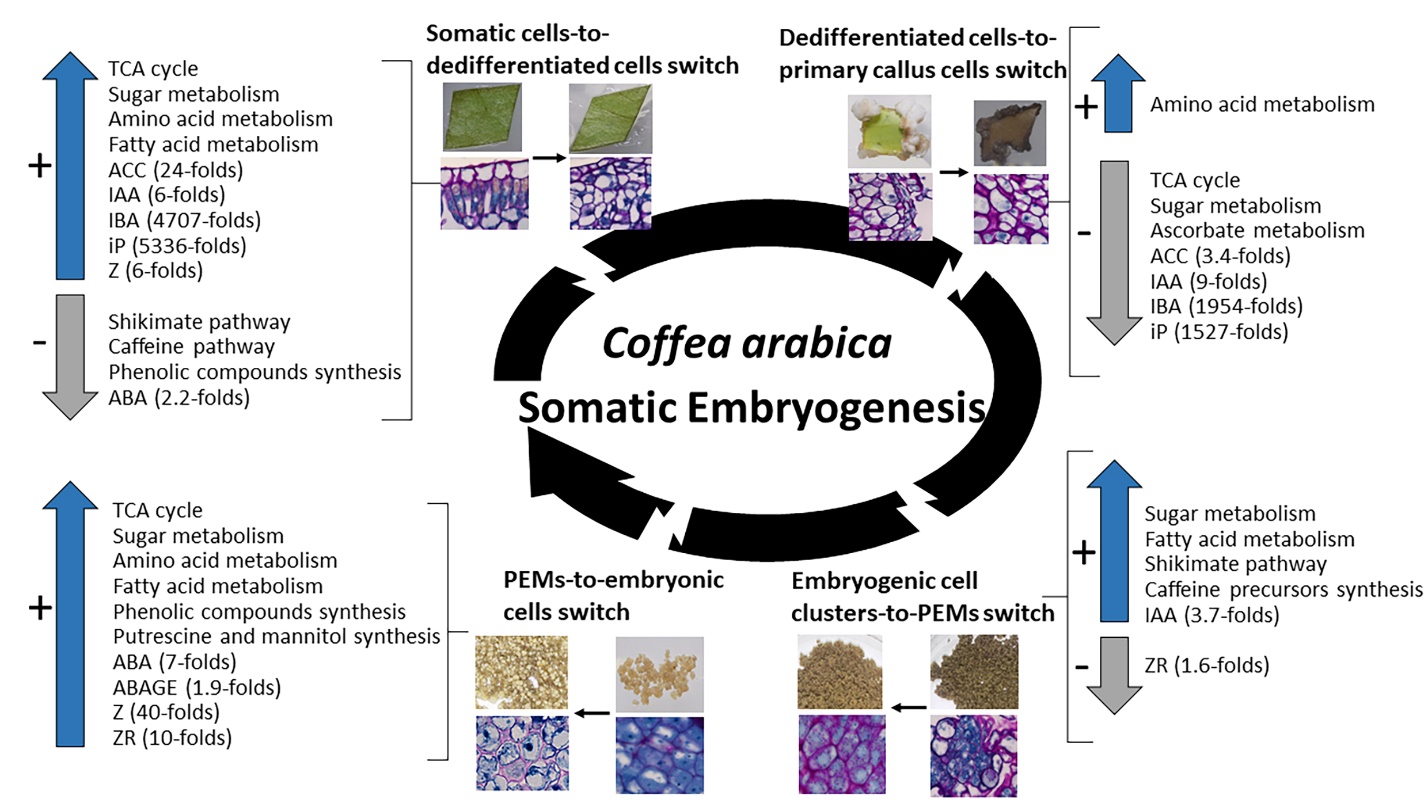
**

**Figure S2.** Metabolic pathways and hormone dynamics during the four main developmental phase switches. The four phase switches are: the leaf explant-to-dedifferentiated cell switch occurring after one week in induction medium, the dedifferentiated cells-to-established primary callus cells switch occurring three months after induction, the embryogenic cell clusters-to-pro-embryogenic masses (PEMs) switch occurring after one week in redifferentiation medium, and the PEMs-to-embryonic cells switch occurring after four weeks in redifferentiation medium. Metabolic pathways were identified based on an enrichment in significantly over-accumulated and under-accumulated metabolites, i.e., Z-score > 1 and Z-score < −1, respectively. All presented hormones differ significantly in their levels according to a two-by-two Wilcoxon mean comparison test (*P* < 0.05). Values represent mean of ratios in hormone levels of independent replicates between a sampled stage and the previous one ± SD (adapted from Awada et al. [58]).
