# Supplementary material for: Global transcriptome profiling reveals differential regulatory, metabolic and hormonal networks during somatic embryogenesis in Coffea arabica
Source: BMC Genomics. 2023 Jan 24;24:41. doi: 10.1186/s12864-022-09098-z (PMC9875526; doi:10.1186/s12864-022-09098-z)
Supplement: Supplementary file 3 — Additional file 3: Figure S3. Characterization of the 12 sampled key developmental stages throughout the Arabica somatic embryogenesis (SE) process at morphological level. [file 12864_2022_9098_MOESM3_ESM.docx]

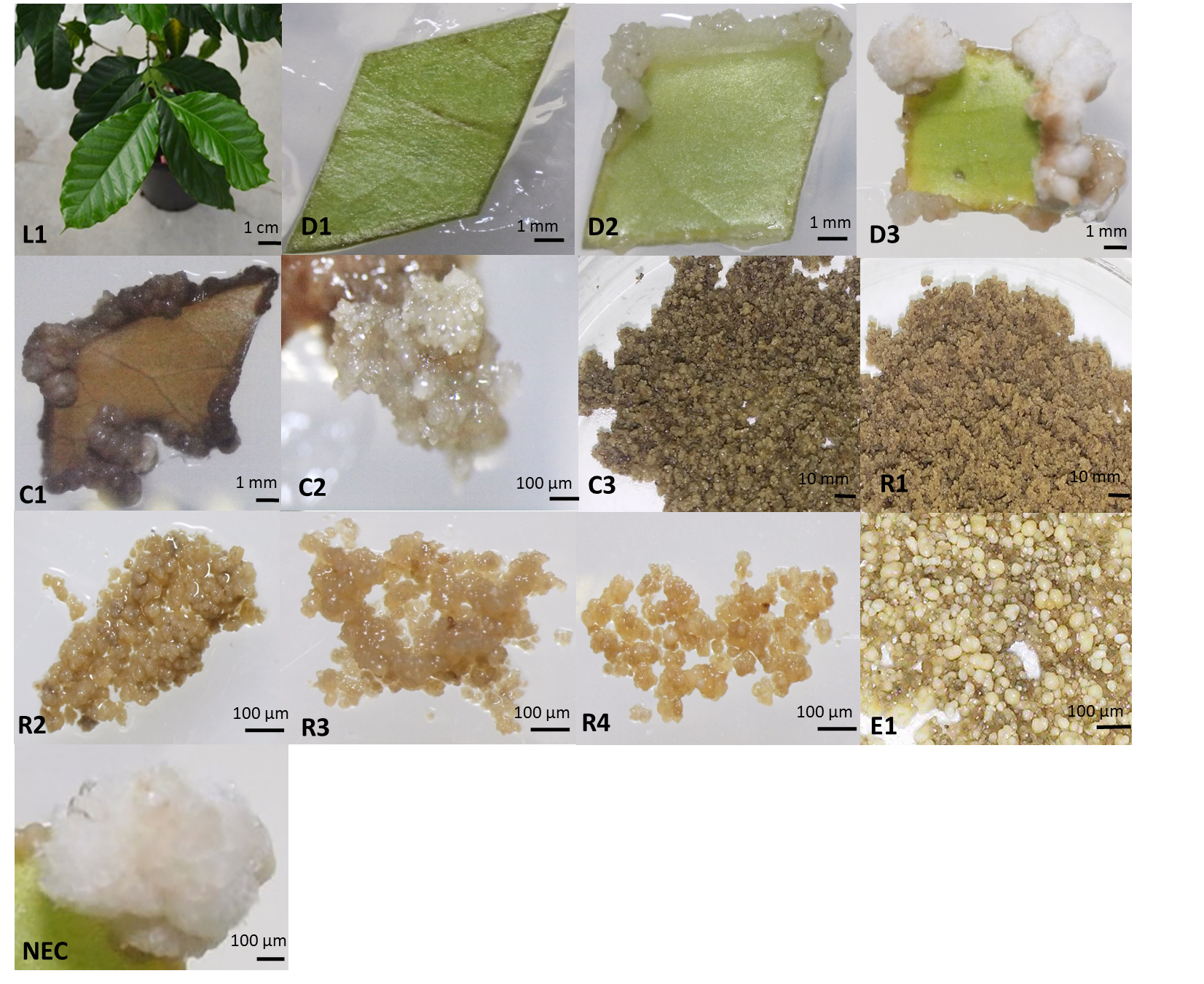


**Figure S3.** Characterization of the 12 sampled key developmental stages throughout the Arabica somatic embryogenesis (SE) process at morphological level. Developmental stages correspond to: leaves from greenhouse plants i.e. explant (L1), explants during dedifferentiation [1 week (D1), 2 weeks (D2), 5 weeks (D3)], compact primary callus obtained 3 months after induction (C1), embryogenic callus obtained 7 months after induction (C2), established cell clusters obtained after 4 months in liquid proliferation medium (C3), pro-embryogenic masses [1 week in redifferentiation medium after auxin withdrawal (R1), 24 h in redifferentiation medium after reducing cell density (R2), 72 h (R3), 10 days (R4)] and globular embryos (E1) obtained after 3 weeks of culture. An additional stage, the non-embryogenic callus (NEC), was obtained using the same culture conditions as for the embryogenic callus (C2) and was sampled at the same time. Phenotyping images were taken using an Olympus E-5 digital camera mounted on an Olympus SZX7 stereomicroscope. The stereomicroscope was not used for (L1). Adapted from Awada et al. [58].
