# Supplementary material for: Global transcriptome profiling reveals differential regulatory, metabolic and hormonal networks during somatic embryogenesis in Coffea arabica
Source: BMC Genomics. 2023 Jan 24;24:41. doi: 10.1186/s12864-022-09098-z (PMC9875526; doi:10.1186/s12864-022-09098-z)
Supplement: Supplementary file 4 — Additional file 4: Figure S4. RT-qPCR verification of selected genes in five SE developmental stages L1, C1, C2, C3, and E1. [file 12864_2022_9098_MOESM4_ESM.docx]

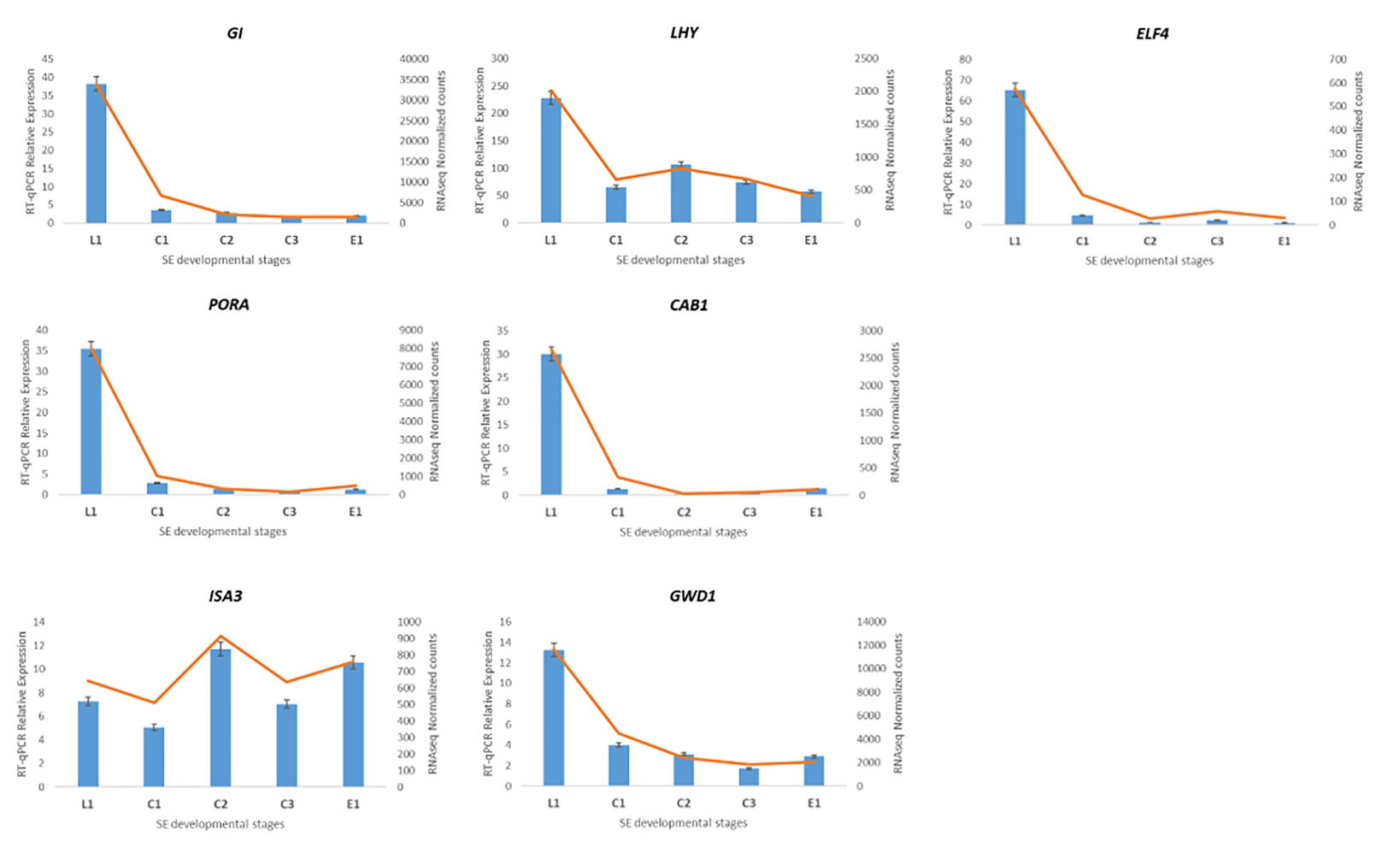


**Figure S4.** RT-qPCR verification of selected genes in five SE developmental stages L1, C1, C2, C3, and E1. The data of polylines is derived from the RNAseq normalized counts of each gene while the data of bar plots is derived from the relative expression levels obtained by RT-qPCR. RT-qPCR data were normalized to *24S* and *PP2A* reference genes and error bars represent ± s.d. (n = 3). The statistical differences were analyzed by ANOVA based on Fisher’s LSD (*P* < 0.05). No statistical differences were noted. L1: leaf; C1: primary callus; C2: embryogenic callus; C3: embryogenic cell clusters; E1: globular embryo.
